# Supplementary material for: Identification and drug-induced reversion of molecular signatures of Alzheimer’s disease onset and progression in AppNL-G-F, AppNL-F, and 3xTg-AD mouse models
Source: Genome Med. 2021 Oct 26;13:168. doi: 10.1186/s13073-021-00983-y (PMC8547095; doi:10.1186/s13073-021-00983-y)
Supplement: Supplementary file 1 — Additional file 1. File including all supplementary figures cited in the text (FigS1-S10), the Table S7 and legends to all the supplementary tables (Table S1-S6) included in the Additional File 2. [file 13073_2021_983_MOESM1_ESM.pdf]

Additional File 1

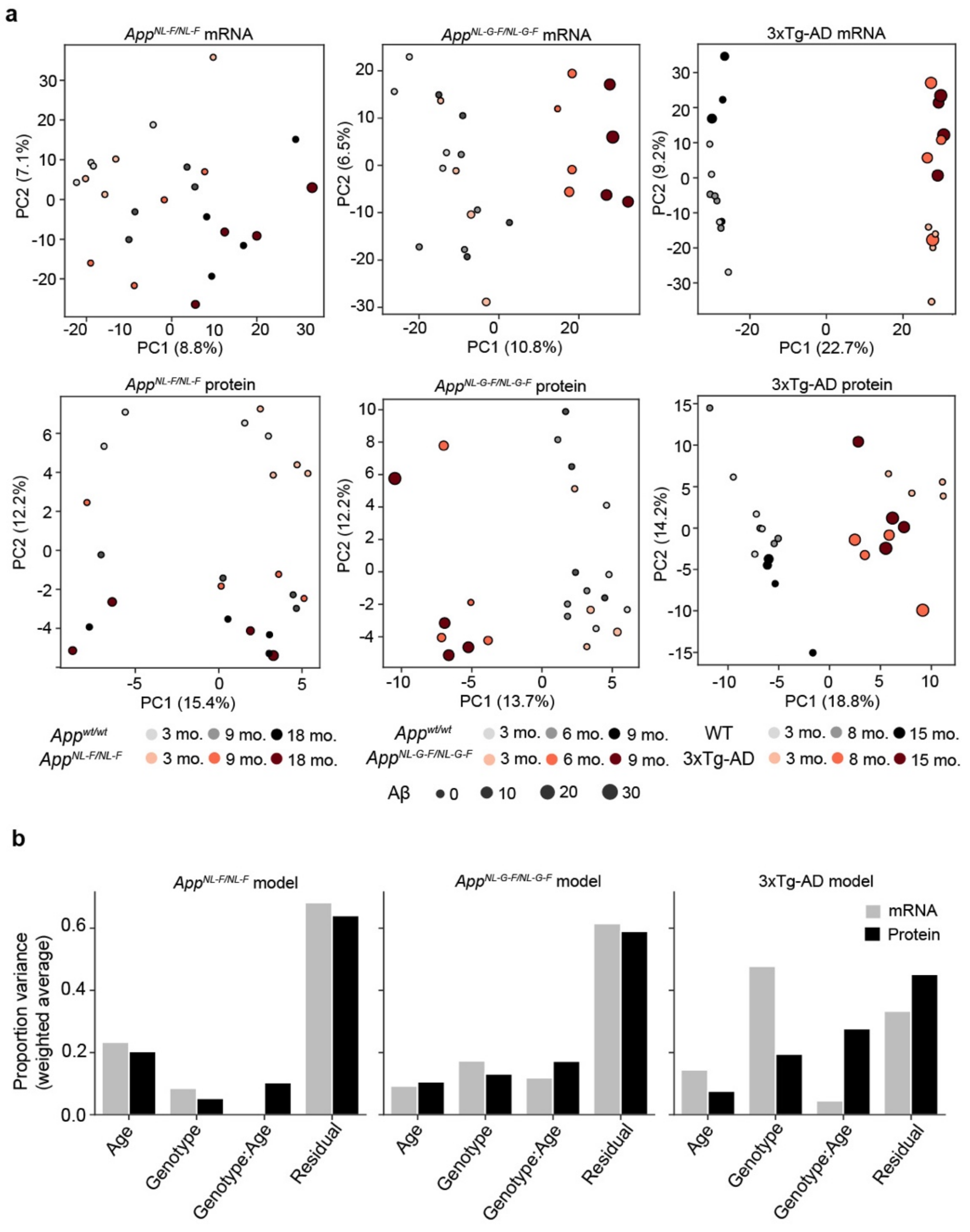

Fig S1. Principal Component Analysis (PCA) of hippocampus gene expression influenced by age and genotype. (a) PCA plot showing distribution of samples corresponding to the *App*$^{NL-F}$ and *App*$^{NL-G-F}$ models and their respective *App*^{wt} controls (gray colors) at different ages (left panel). On the right panel, the PCA plot shows the separation for 3xTg-AD mice and the corresponding wild-type controls (C57BL6/129SvJ; gray colours). Size of the dot is proportional to the quantification of Aβ for each of the samples. **(b)** Principal Variance Component Analysis

(PVCA) showing which sources of variability are most prominent in each dataset after adjustment for batch (mRNA) or random effects (protein).

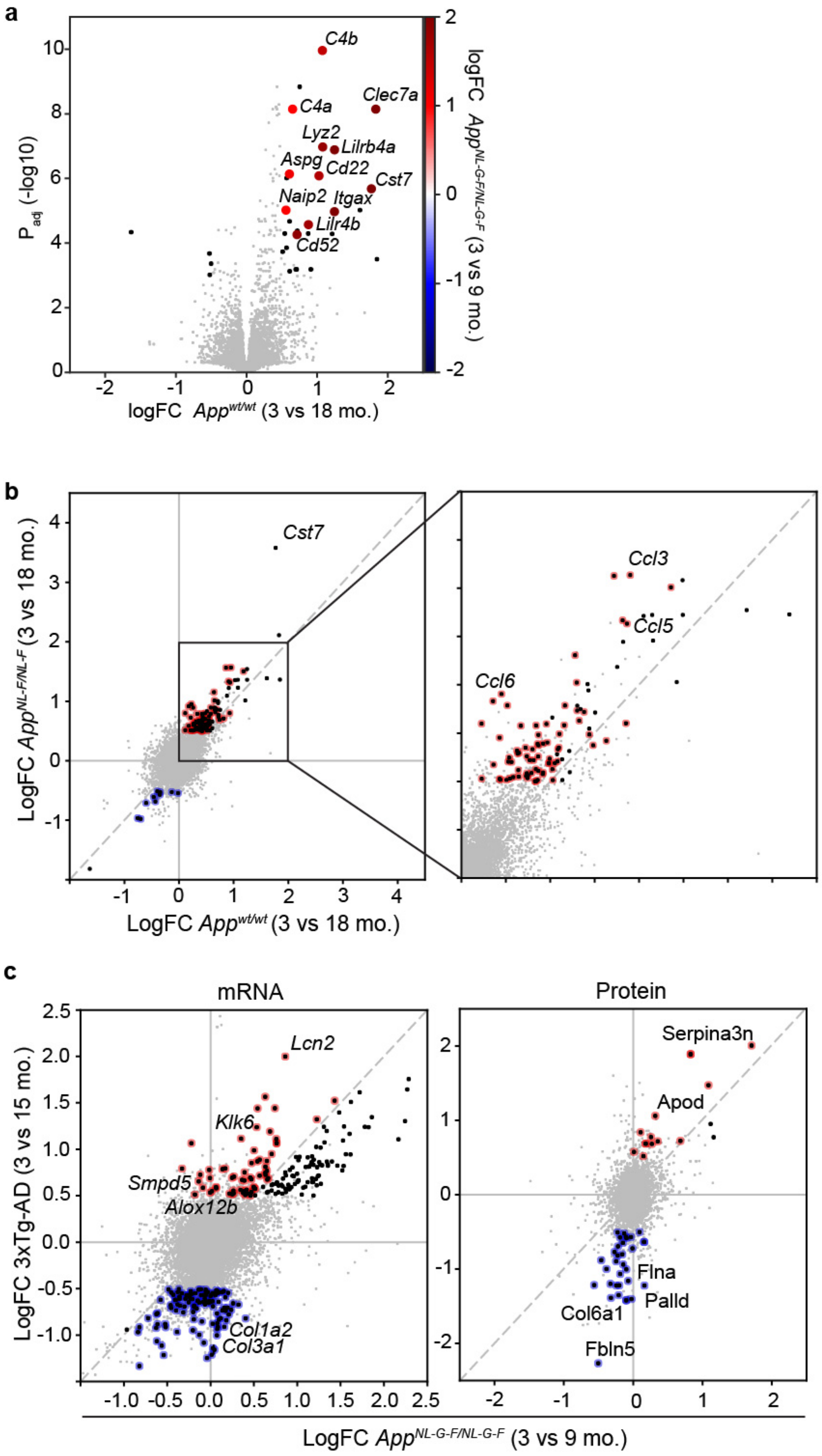

Fig S2. (a) A Volcano plot reporting the gene expression logarithmic fold-change (logFC; X-axis) and the adjusted p-value (*P*adj; Y-axis) when analyzing the 3- vs. 18-month-old (mo.) App^{wt} mice comparison (n=4). Genes that show a higher fold-change in the 3- vs. 9-mo. $App^{NL\text{-}G\text{-}F}$ mice comparison are highlighted using a color gradient. **(b)** Scatter plot representing fold-changes of the 3- vs. 18-mo. App^{wt} mice (X-axis) compared with the fold-changes in the 3- vs. 18-mo. $App^{NL\text{-}F}$ mice (Y-axis). Black dots are used to represent significantly up-/down-regulated genes in the $App^{NL\text{-}F}$ model (Abs. logFC>0.5, FDR < 5%), while colored dots identify genes significantly up- (red) or down-regulated (blue) only in the $App^{NL\text{-}F}$ comparison but not in the App^{wt} comparison (n=4). **(c)** Scatter plot comparing Log fold-changes of genes (left panel) and proteins (right panel) of $App^{NL\text{-}G\text{-}F}$ mice comparing 3- vs. 9-mo. ages (X-axis) and 3xTg-AD mice comparing 3- vs. 15-mo. ages (Y-axis). Black dots are used to represent significant changes in the 3xTg-AD model (Abs. logFC>0.5, FDR<5%). Red (upregulated) and blue (downregulated) dots represent genes/proteins with an absolute logFC higher for the 3- vs. 15-mo 3xTg-AD. comparison than in the comparison of 3- vs. 9-mo. $App^{NL\text{-}G\text{-}F}$ mice (n=4).

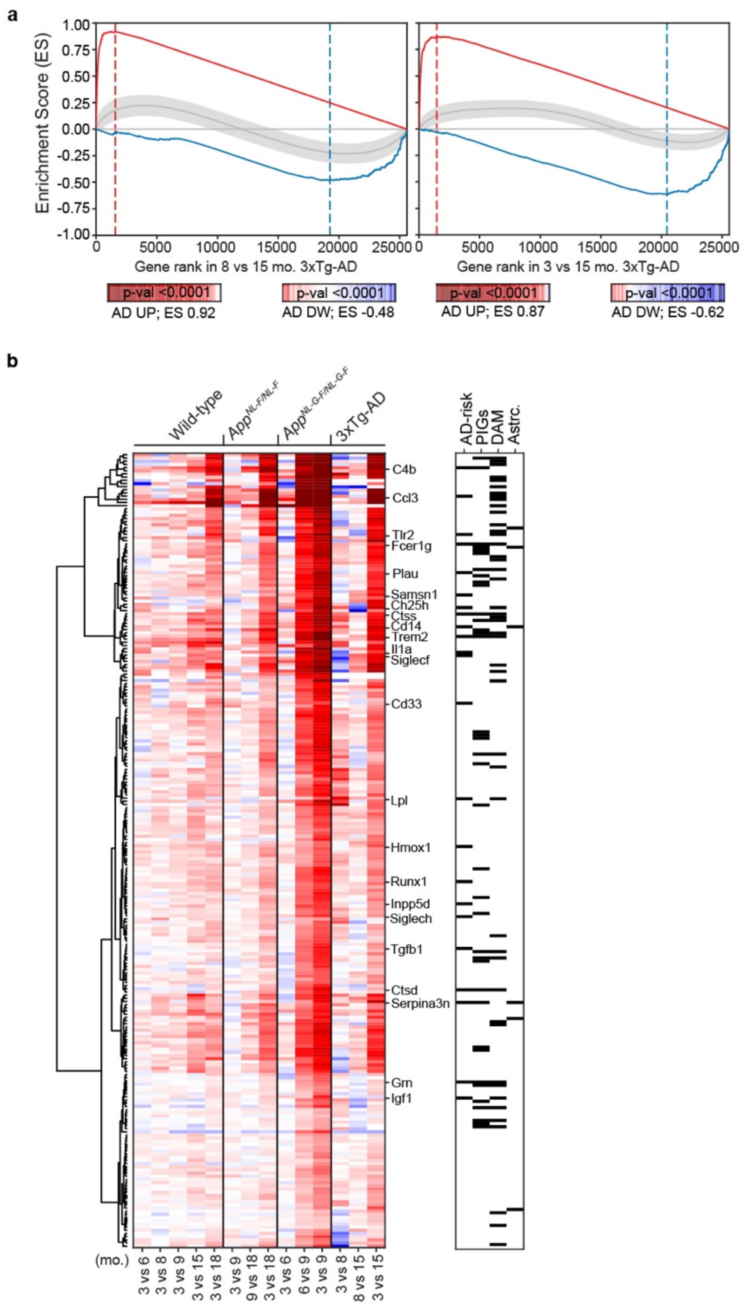

Fig S3. Analysis of the *AD signatures*. (a) Validation of the *AD signatures* in 3xTg-AD mice. In the X-axis, genes are ranked by their differential expression in 15-mo. 3xTg-AD mice compared to earlier time points (8 and 3 mo.). The Y-axis represents the running Enrichment Score (ES) computed for the *AD-UP signature* (red) and *AD-DW signature* (blue), which were derived from *App*$^{NL\text{-}G\text{-}F}$ mice and are recapitulated in the 3xTg-AD mouse model. RNAseq data were obtained from n=4 mice per condition. **(b)** Heatmap showing the progression of transcriptional changes captured by the *AD-UP signature* across models. Each gene is represented by a row of colored tiles, the color representing the expression level for the indicated condition (red, upregulated; blue, downregulated). On the right, black squares indicate whether the gene is present in a list of

genes linked to AD risk (AD-risk), genes detected as proximal to amyloid plaques (Plaque-induced Genes, PIG), or in a list of genes identifying Disease-associated microglia (DAM). For the AD-risk genes, name is indicated next to the heat map.

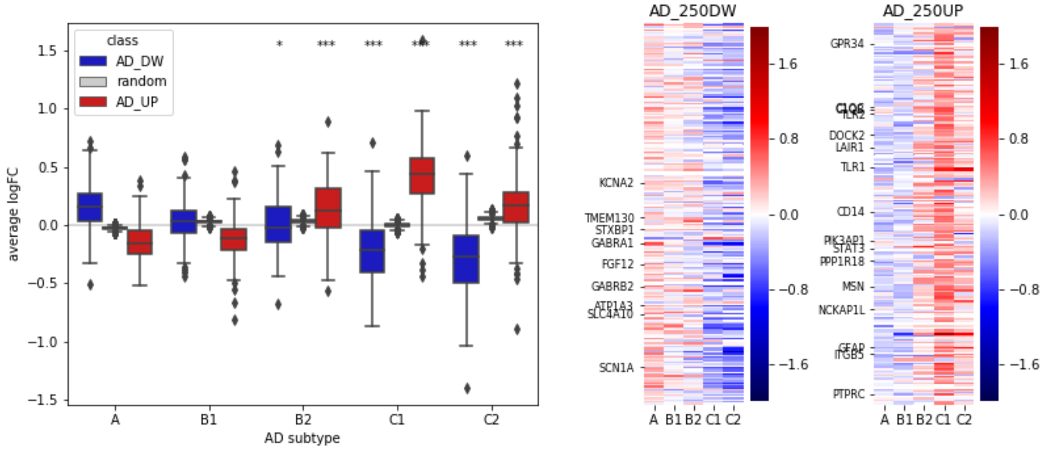

Fig S4. Matching AD signatures derived from *App* knock-in mice to human derived MSBB-AD subtype signatures. The boxplots represent the change in expression of *App*$^{NL\text{-}G\text{-}F}$ AD-UP and AD-DW signature genes in the parahippocampal gyrus of patients of five AD-subtypes compared to normal controls. Asterisks represent the empirical p-value of the observed average log fold change (10,000 permutations). The heatmaps show the log fold change of individual AD signature genes in human MSBB-AD subtypes and highlights key regulator genes identified in patients.

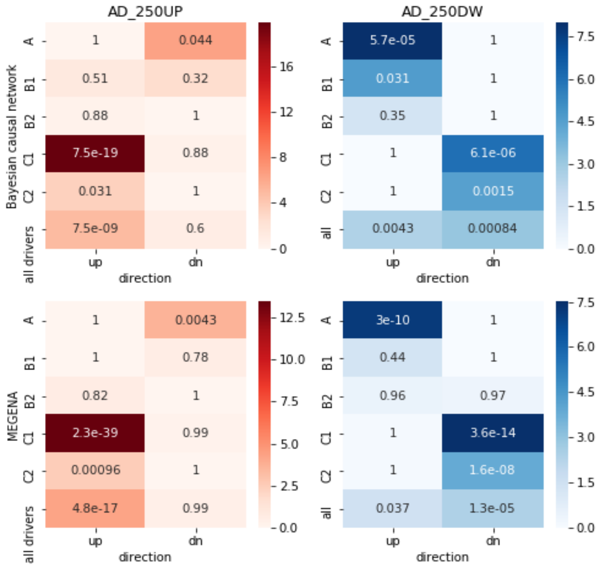

Fig S5. Enrichment of up- and down-regulated MSBB-AD Key Network Regulators (KNRs) found in the *App*$^{NL\text{-}G\text{-}F}$ derived AD signatures.

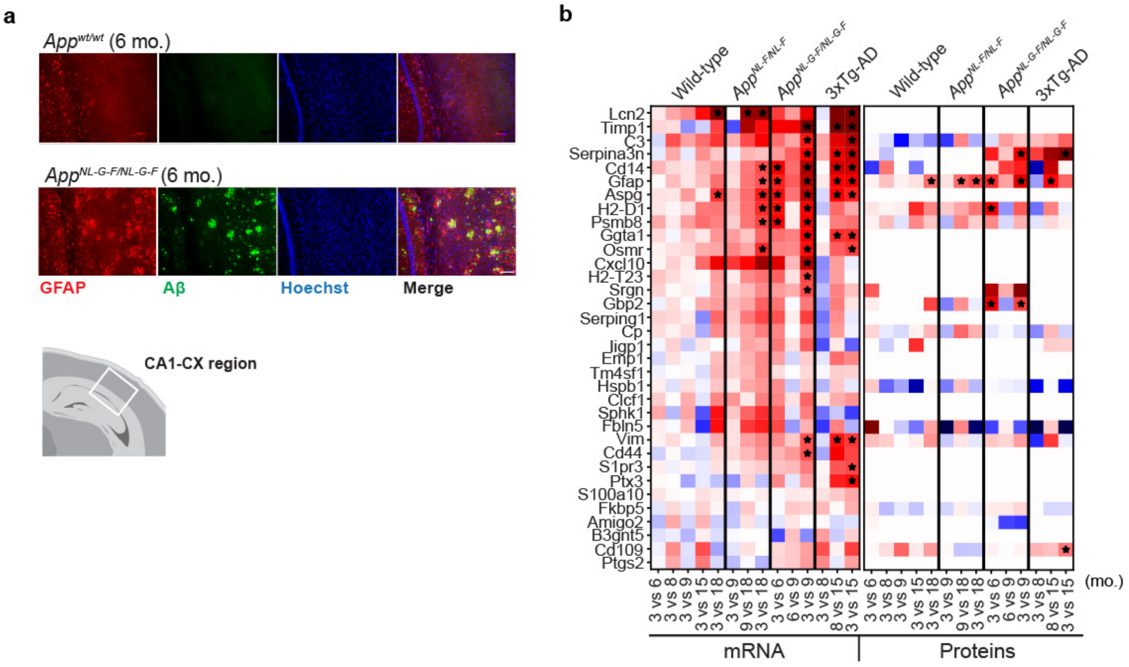

Fig S6. Analysis of the astrocytic signature. (a) Representative microphotographs of the CA1-CX region of slices of the brain of 6-mo. *App*^{wt} and *App*$^{NL\text{-}G\text{-}F}$ mice stained with an anti-GFAP antibody (red), an anti-Aβ antibody (green) and Hoechst dye (blue). Scale bar represents 100 μm. N=3. **(b)** A heatmap showing the LogFC values corresponding to the gene expression and protein analysis of the 34 genes that constitute the *AD-astrocytosis* signature as defined in the main text. Each gene is represented by a row of colored tiles, the color representing the fold change in expression for the indicated condition (red, upregulated; blue, downregulated). Stars indicate statistically significant changes (|logFC|>0.5 for mRNA, |logFC|>0.25 for protein; FDR<5%).

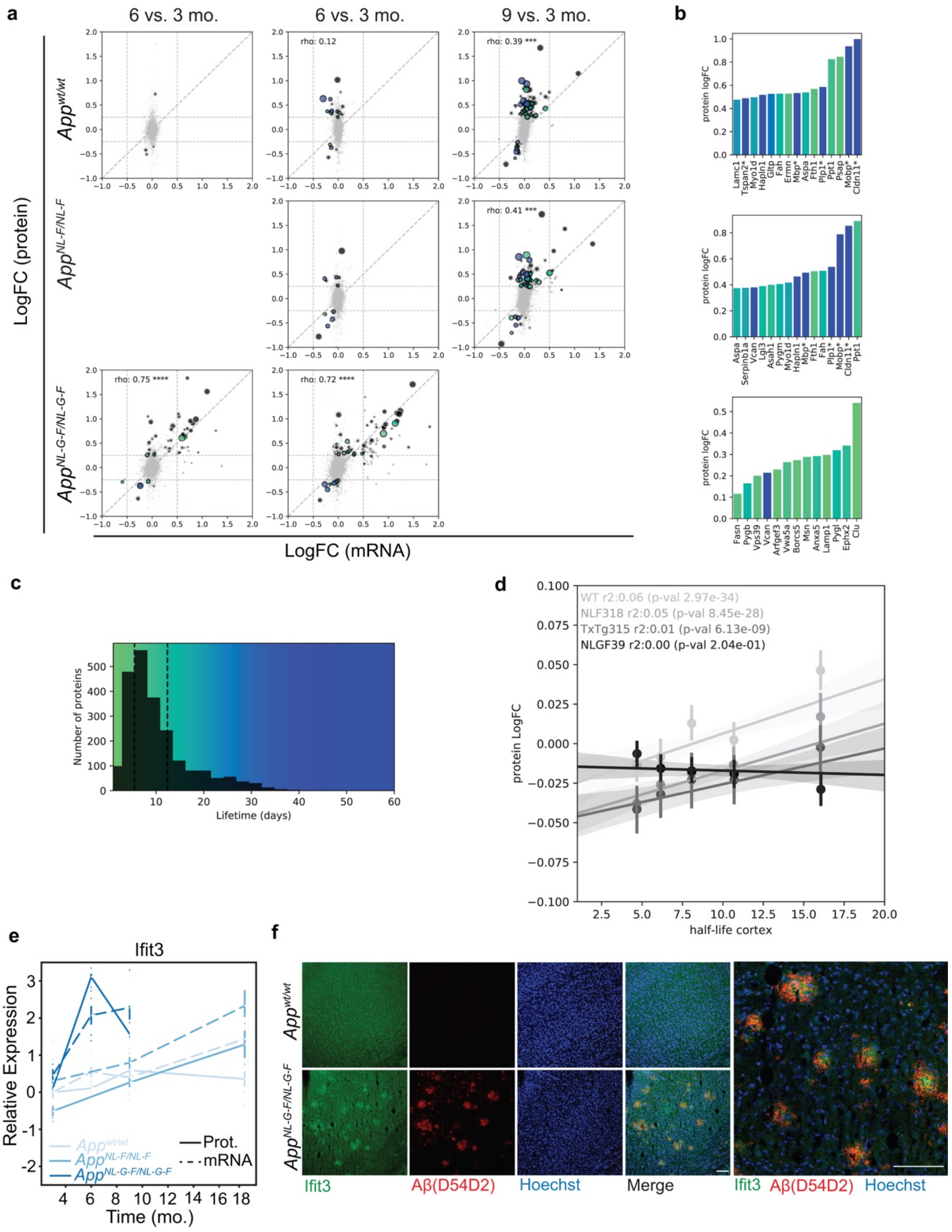

Fig S7. Analysis of mRNA and protein correlation in the different comparisons. (a) Scatter plot depicting the Logarithm of the Fold-Change (LogFC) of mRNA (X-axis) and protein (Y-axis) for the comparison of 6-, 9-, and 18-mo. with respect to 3-mo. App^{wt}, $App^{NL\text{-}F}$, and $App^{NL\text{-}G\text{-}F}$ mouse models. Proteins whose LogFC(protein)>0.25 and their corresponding LogFC(mRNA)>0.5 at a 5% FDR threshold are highlighted. Dot color indicates the half-life of the proteins in cortex

homogenates, as defined by (doi: 10.1038/s41467-018-06519-0). Dot size is proportional to the negative logarithm of the adjusted p-value of protein changes. **(b)** Bar plot representation of the strongest discordant changes in protein abundance with estimated protein lifetimes represented as a color scale. Myelin proteins (highlighted with an asterisk) tend to accumulate in aged mice, together with other long-lived proteins. **(c)** Distribution of the estimated protein lifetime in our dataset and color scale used in **a** and **b**. Dashed lines indicate the upper and lower quartiles of the distribution (12.5 and 5.5 days, respectively). **(d)** Association between discordant changes in protein abundance with respect to protein lifetimes. The lines show the fit of a linear regression model with points indicating the average protein LogFC of 5 equally sized bins, with error bars indicating the 95% confidence interval. Proteins showing concordant changes with mRNA were excluded from this analysis. The coefficients of determination (r2) are very modest but significant in aged mice (App^{wt}, $App^{NL\text{-}F}$ and 3xTg-AD) but not in younger and more aggressive AD mouse models ($App^{NL\text{-}G\text{-}F}$). **(e)** Ifit3 protein (continuous line) and mRNA (dashed line) levels at different time points relative to the 3 mo. App^{wt} model are shown for the $App^{NL\text{-}G\text{-}F}$ (strong blue), $App^{NL\text{-}F}$ (medium blue) and App^{wt} (corresponding to the $App^{NL\text{-}F}$ model; light blue) mice. N=4. **(f)** Representative micrographs of brain slices of 6-mo. $App^{NL\text{-}G\text{-}F}$ mice stained with an anti-Ifit3 antibody (green), the anti-Aβ antibody D54D2 (red) and Hoechst dye (blue). Scale bars represent 100 µm (n=3).

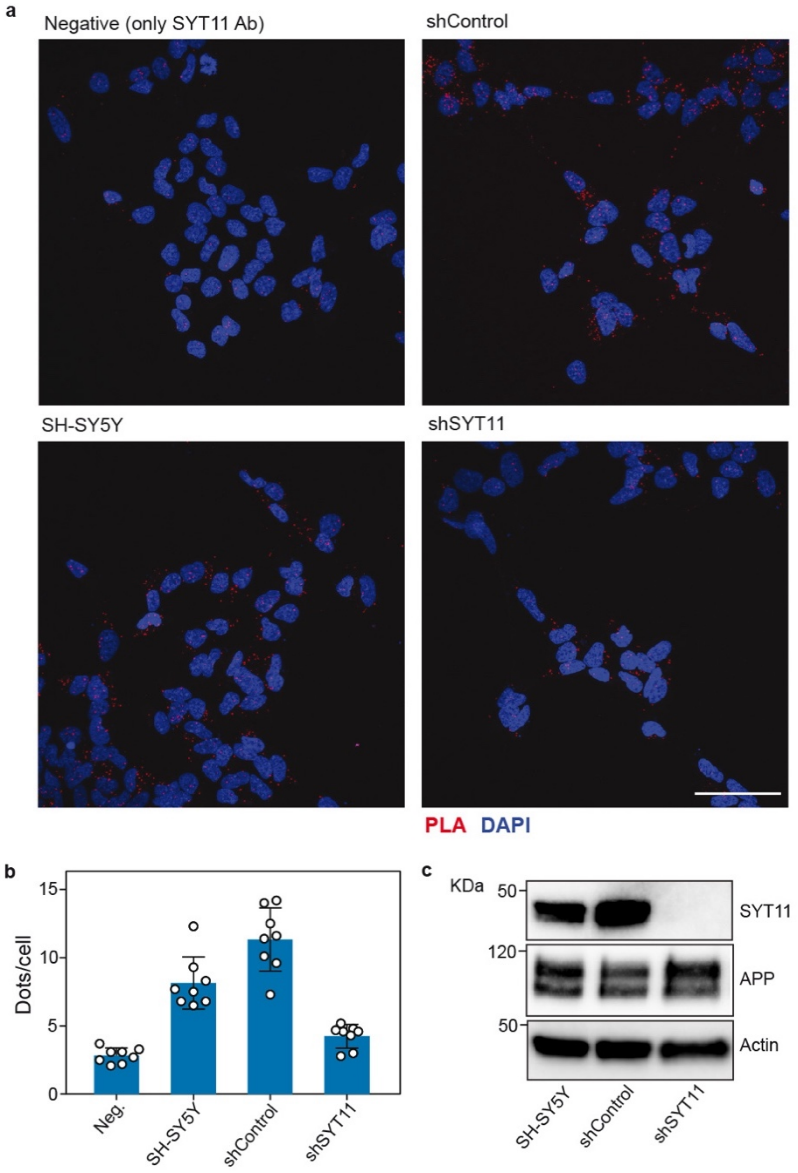

Fig S8. PLA analysis of SYT11 and App interaction in SH-SY5Y cells. (a) Representative micrographs of differentiated SH-SY5Y cells that were treated only with anti-SYT11 antibody (negative control) or both anti-SYT11 and anti-App antibody (clone 6E10; SH-SY5Y). SH-SY5Y stably expressing a control shRNA (shControl) and an shRNA targeting SYT11 (shSYT11) were also used. Bar indicates 50 µm. **(b)** Quantification of 8 different fields corresponding to the experiment shown in (a). PLA positive dots (red) were quantified and expressed as dots per number of cells. Cell nuclei are stained with DAPI (blue) to identify individual cells. Mean±SD are shown, n=8. **(c)** Western blot of differentiated, non-transduced, SH-SY5Y cells (SH-SY5Y) or cells expressing control shRNA (shControl) or a shRNA targeting SYT11 (shSYT11). In all panels, a representative experiment out of three independent experiments is shown.

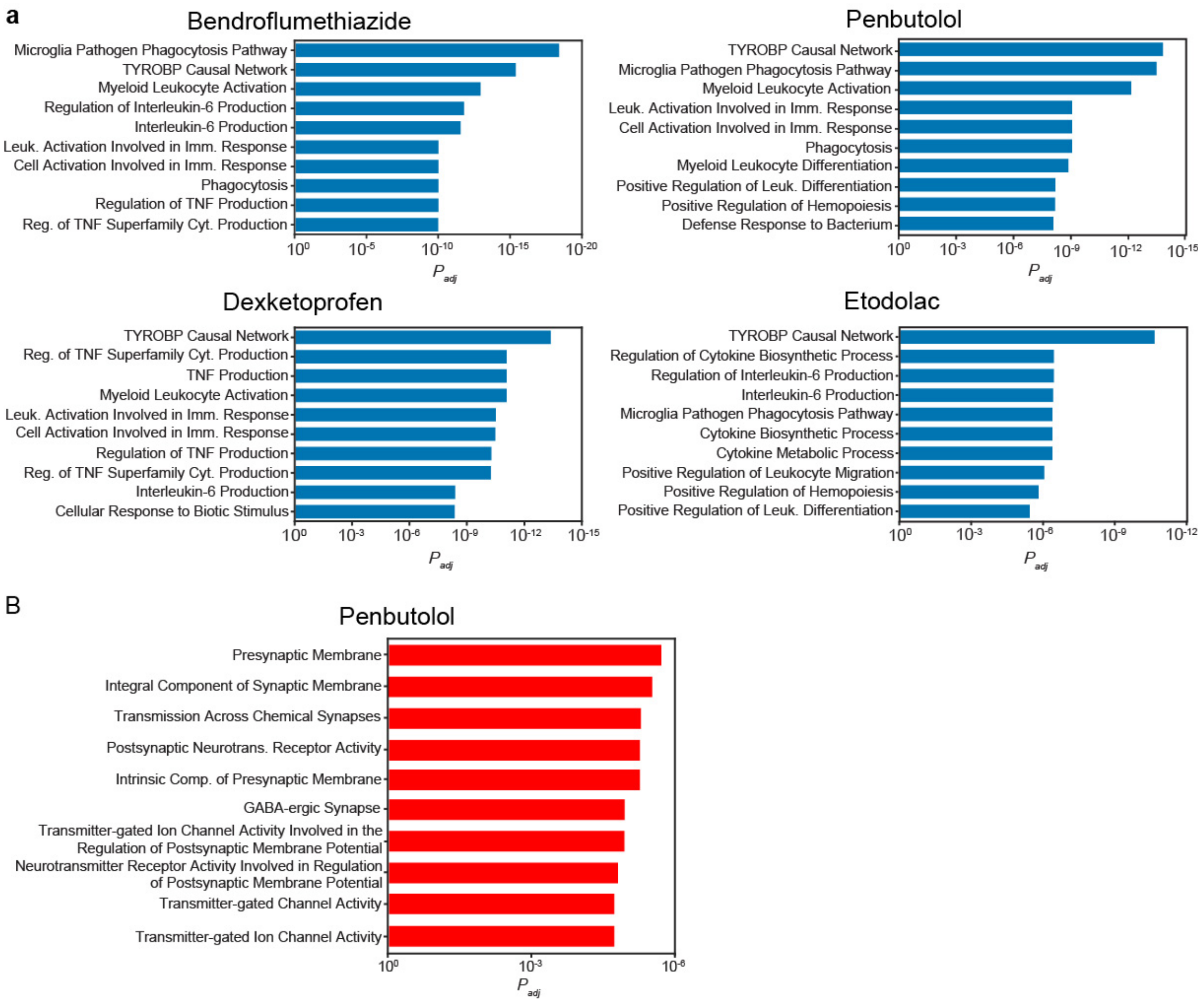

Fig S9. Analysis of the signature reversion in treated mice. Functional enrichment analysis of the leading-edge of the **(a)** *AD-UP signature* reversion (blue) or **(b)** *AD-DW signature* reversion (red) induced by the different treatments. The bars show the adjusted p-value of the 10 most overrepresented biological pathways. RNAseq data were obtained from n=4 mice per condition.

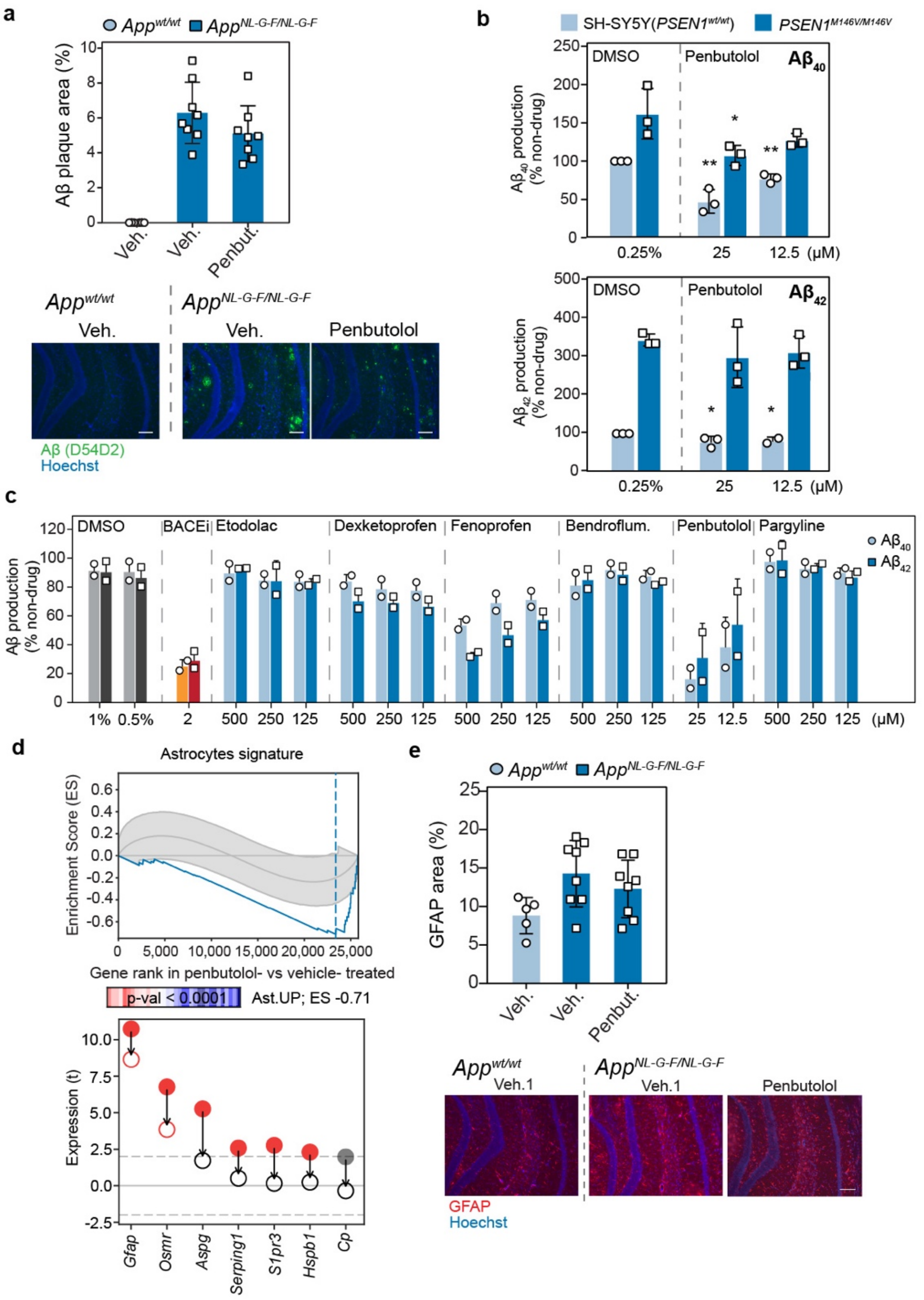

Fig S10. **Penbutolol prevents Aβ accumulation and astrocytosis. (a)** Percentage of Aβ-positive area measured in brain sections of 6-mo penbutolol- and vehicle-treated *App*^{wt} (circles) and *App*$^{NL\text{-}G\text{-}F}$ (squares) mice. Mean±SD are shown (n= 3-4 for each condition). Below, representative microphotographs of the DG-CA1 region of the hippocampus stained with an anti-Aβ antibody (green) and Hoechst dye (blue) are shown. Scale bars represent 100 μm. **(b)** Effect of penbutolol in a cellular AD model. Normalized $Aβ_{40}$ and $Aβ_{42}$ secretion in differentiated wild-type (*PSEN1*^{wt}) or mutated (*PSEN1*$^{M146V/M146V}$) SH-SY5Y cells treated with the indicated

concentration of penbutolol. Mean ± SD of 3 independent experiments are shown. Unpaired Student's t-test (** P < 0.01, * P < 0.05). **(c)** Aβ production in 7PA2 cells. Normalized $Aβ_{40}$ and $Aβ_{42}$ secretion in 7PA2 cells treated with the indicated compounds. Percentage of production of $Aβ_{40}$ (circle, light colors) and $Aβ_{42}$ (square, dark colors) compared with cells treated in the absence of drug. DMSO (gray) and a BACE1 inhibitor (BACEi; AZD3839) are used as controls. Mean ± SD of two independent experiments are shown. **(d)** Reversion of genes belonging to the previously described *astrocytosis signature*. As in Figure 5, the top panel shows a graphic representation of the Enrichment Score (ES). In the X-axis, genes are ranked by their differential expression in the comparison of penbutolol- vs. vehicle-treated App^{NL-G-F} mice. The Y-axis represents the running ES computed for the *astrocytosis signature*, which tends to be downregulated by penbutolol treatment in App^{NL-G-F} mice. On the bottom panel, example genes that are up- (red) or down-regulated (blue) (t-score) in the vehicle-treated App^{NL-G-F} vs. vehicle-treated App^{wt} comparison (bold dots) or in the penbutolol-treated App^{NL-G-F} vs. vehicle-treated App^{wt} comparison (empty dots). RNAseq data were obtained from n=4 mice per condition. **(e)** As in **(a)**, percentage of GFAP-positive staining area was measured. Mean±SD are shown (n= 3-4 for each condition). Representative images of the DG-CA1 region stained with an anti-GFAP antibody (red) and Hoechst dye are shown on the bottom.

Table S1. Genes and proteins associated with healthy aging. mRNA (|logFC|>0.5; FDR<5%) and proteins (|logFC|>0.25; FDR<5%) significantly up- or down-regulated when comparing 3- vs. 18-mo. App^{wt} mice are listed. For each gene, when available, mRNA and protein logFC and FDR are provided. Genes simultaneously found significant at protein and mRNA level are highlighted in gray.

Table S2. Genes and proteins specifically associated with AD pathology in aged App^{NL-F} mice. mRNA (absolute logFC>0.5; FDR<5%) and proteins (absolute logFC>0.25; FDR<5%) significantly up- or down-regulated when comparing 3- vs. 18-mo. App^{NL-F} mice but not found significantly changed in App^{wt} ageing (see Table S1) are listed. For each gene, when available, mRNA and protein logFC and FDR are provided. Genes simultaneously found significant at protein and mRNA level are highlighted in gray.

Table S3. Genes and proteins specifically associated with 3xTg-AD pathology. mRNA (absolute logFC>0.5; FDR<5%) and proteins (absolute logFC>0.25; FDR<5%) significantly up- or down-regulated when comparing 3- vs. 15-mo. 3xTg-AD mice are listed (see Fig. S2c). Absolute logFC and adjusted p-values for 3- vs. 9-mo. App^{NL-G-F} comparison and the 3- vs. 15-mo. wild-type comparison are also shown.

Table S4. AD-signatures. Absolute logFC, adjusted p-values and FDR for mRNA levels of genes identified in the AD-UP and AD-DW signatures. Columns AD-risk, DAM and PIGs indicate whether these genes are included in any given group of genes (*True*, they belong; *False*, they do not). When available, values for the quantified protein are also provided.

Table S5. Result of the virtual signature-based screening of compounds. This table shows the detailed scores of the signature-based prioritization of compounds. Compounds selected in at least one of the five queries (Q1-Q5) are shown (8,250 in total). Compounds are identified by

InChIKey (ik) and an internal identifier (id); ik is used to rank compounds by default. Molecular weight (mw), Lipinski's Rule-of-5 violations (ro5), chemical beauty (qed) are given for every compound. In addition, the source of the compound is specified (1/0); alzf: available in AlzForum (i.e., a drug with previously tested against AD), drug: available in DrugBank, pres: part of the Prestwick library, and irb: part of an IRB Barcelona proprietary library. In blue (1/0), we highlight the query (q1-q5) where the compound was a hit; please note that a 0-value in this column does not mean that the compound is not relevant to that query but rather that it was not among the top-scoring hits. The following columns correspond to BBBP, BACE and Aβ activity predictions, based on supervised machine learning (bbbp, bace, ab42, ab40, abratio). In addition, we list putative targets of the molecules (targets), together with a probability score assigned with a simple ligand-based similarity-ensemble approach (tm) {Keiser, 2007 #112}; the column targets_agg simply adds these scores, thereby quantifying the potential promiscuity of the molecule. The remaining columns correspond to the signature-based search. They are organised in colors, red denoting similarity searches against known AD drugs, green transcriptional signature matching, and yellow searches based on the interaction or proximity to putative AD targets. Values shown in these columns correspond to -log10 p-values; these p-values were calculated empirically over the full CC universe (>800k molecules). (Red) Similarity to AD drugs is prefixed with sim_ad. For each molecule, similar AD drugs are listed in sim_ad_drugs (separated by | and separating chemical similarity (sim) and cell-based (phenotypic) similarity (ph) inside the parenthesis); an aggregated score for the overall similarity to AD drugs is given in sim_ad_drugs_agg. Next, similarities are calculated for different AD drug categories, namely amyloid, cholesterol, cholinergic, inflammation, tau, other_neurotrans[mitters], and other. (Green) Transcriptional signature matching is split in five priorities (p4-p0); AD signatures (sigs) identified in human (p4, prefixed jager {Mostafavi, 2018 #93}) and our signatures identified in mice (p3, prefixed sbnb, for Structural Bioinformatics & Network Biology) being the categories with top priority, followed by signatures identified in AD cell models (p2, prefixed sbnb_cells), signatures identified in GEO (p1, prefixed geo) and, finally, signatures related to known AD drugs available from LINCS (p0, prefixed with drug InChIKey; in addition, this lowest-priority column contains signatures of proteomics experiments, which we consider less reliable for matching with the transcriptional profiles of the molecules). Columns pX_sigs list the particular signatures matched, together with a connectivity score (con) and a guilt-by-association score (gba), used to relate molecules (based on similarity) to their closest analogs in the LINCS repository (CC space D1) from where transcriptional signatures are available. Columns pX_sigs_agg give an aggregated score for the signature-based search. (Yellow) Compound interaction with putative AD relevant targets. These columns correspond to targets relevant to AD (extracted from OpenTargets, suffixed rational) or targets with a putative association with AD based on LINCS L1000 shRNA experiments (suffixed sh_lincs and following the pX scheme explained above). The relevance of the target to AD is denoted ad:score, corresponding to the OpenTargets score. Interaction of the small molecule with its target is noted with the ligand-based approach (tm); the target of the molecule and the AD target are related with a proximity (prox) score based on the network-based proximity measures developed for the CC (C3-5 levels).

Table S6. Transcriptional reversion of AD signatures. List of genes that comprise the leading edge of the transcriptional reversion of the *AD-UP* and *AD-DW* signatures induced by the different

treatments. The z-score of the differential expression analysis of treated and untreated *App*$^{NL\text{-}G\text{-}F}$ mice vs. *App*^{wt} is provided for each gene. The gene rank of the treated vs. untreated comparison is provided in bins of 1000 (5 = top 1000, 4 = top 2000, 3 = top 3000, 2 = top 4000, 1 = top 5000). Negative signs denote down-regulation. Additional annotations highlight those genes that have been previously related to Alzheimer's disease or that are up-regulated in disease associated microglia or reactive astrocytosis.

Table S7. Sequences for mouse genotyping by PCR and shRNA target sequence

**Primer**	**Model**	**Sequence (5'-3')**
**App_F**	3xTg-AD	AGGACTGACCACTCGACCAG
**App_R**	3xTg-AD	CGGGGGTCTAGTTCTGCAT
**Tau_F**	3xTg-AD	GGGGGACAGGAAAGATCAG
**Tau_R**	3xTg-AD	GTGACCAGCAGCTTCGTCTT
**IL2_F (internal control)**	3xTg-AD	CTAGGCCACAGAATTGAAAGATCT
**IL2_R (internal control)**	3xTg-AD	GTAGGTGGAAATTCTAGCATCATCC
**App_WT_F**	*App*$^{NL\text{-}F}$; *App*$^{NL\text{-}G\text{-}F}$	ATCTCGGAAGTGAAGATG
**App_WT_R**	*App*$^{NL\text{-}F}$; *App*$^{NL\text{-}G\text{-}F}$	TGTAGATGAGAACTTAAC
**App_KI_F**	*App*$^{NL\text{-}F}$; *App*$^{NL\text{-}G\text{-}F}$	ATCTCGGAAGTGAATCTA
**App_KI_R**	*App*$^{NL\text{-}F}$; *App*$^{NL\text{-}G\text{-}F}$	CGTATAATGTATGCTATACGAAG
**shControl**	-	CAACAAGATGAAGAGCACCAA
**shSYT11**	-	GCCTCTTATATCTCTGCTCTT
